# Personality may influence reactivity to stress

**DOI:** 10.1186/1751-0759-1-5

**Published:** 2007-03-01

**Authors:** Arnljot Flaa, Øivind Ekeberg, Sverre Erik Kjeldsen, Morten Rostrup

**Affiliations:** 1 Department of Acute Medicine, Ullevaal University Hospital, Oslo, Norway; 2 Department of Cardiology, Ullevaal University Hospital, Oslo, Norway; 3 Cardiovascular and Renal Research Center, Ullevaal University Hospital, Oslo, Norway

## Abstract

**Background:**

Possible mechanisms behind psychophysiological hyperreactivity may be located at a cognitive-emotional level. Several personality traits have been associated with increased cardiovascular reactivity. Subjects with white coat hypertension, which may constitute a kind of hyperreactivity, are found to suppress their emotions and adapt to the surroundings to a larger extent than controls.

We hypothesized in this study that a) stress reactivity is related to personality, and that b) responses to cold pressor test (CPT) and mental stress test (MST) are associated with different personality traits.

**Methods:**

87 men were selected from the 1^st^, 50^th ^and 99^th ^percentile of a blood pressure screening. Cardiovascular and catecholamine responses to MST and CPT were recorded. Fifteen personality traits were assessed using the Karolinska Scale of Personality. Possible independent explanatory predictors for cardiovascular and catecholamine variables at baseline and during stress were analyzed in multiple linear regression analyses using a stepwise forward procedure.

**Results:**

Multiple regression analyses showed that muscular tension (β = 0.298, p = 0.004), irritability (β = 0.282, p = 0.016), detachment (β = 0.272, p = 0.017), psychasthenia (β = 0.234, p = 0.031) and somatic anxiety (β = 0.225, p = 0.046) were significant explanatory variables of reactivity to CPT. During MST, verbal aggression (β = -0.252, 0.031) and detachment (β = 0.253, p = 0.044) were significant predictors of norepinephrine and diastolic blood pressure response, respectively.

Based on KSP-trait quartiles, delta (Δ) systolic (p = 0.025) and Δ diastolic blood pressure (p = 0.003) during MST were related to detachment score, with the highest reactivity in the 4^th ^quartile, while Δ norepinephrine was significantly related to muscular tension (p = 0.033). Δ systolic and Δ diastolic blood pressure responses to CPT were dependent on detachment (p = 0.049 and p = 0.011, respectively) and psychasthenia (p = 0.020 and p = 0.015), while high verbal aggression was associated with lower reactivity measured by Δ norepinephrine (p = 0.037).

**Conclusion:**

The present study indicates that stress reactivity is clearly related to different personality traits, without any single trait being dominant over others. Furthermore, personality seems to have as much, or even more, importance of predicting responses to CPT than responses to MST.

## Background

We have previously demonstrated that blood pressure level at a military draft screening in healthy, young men reflects blood pressure, heart rate and arterial catecholamine reactivity to a mental arithmetic stress test (MST) [1]. This differentiated response pattern was not seen during a cold pressor test (CPT) or an orthostatic test, which are believed to be more physical tests, compared to MST. There are several possible mechanisms behind psychophysiological hyperreactivity, and they can be grouped in three main categories [2].

The first is the cognitive-emotional level. The cortex and the limbic system (brain structures above hypothalamus) are responsible for temperament and personality, and several personality traits have been associated with increased cardiovascular reactivity [3,4]. White coat hypertension is a kind of hyperreactivity, and is related to a larger degree of suppressing own emotions and adaptation to the surroundings [5]. A recent study, exposing low- and high systolic blood pressure reactors to a Stroop stress test while performing functional MRI, revealed increased activity of the posterior cingulate cortex in the high reactors [6].

A second possibility is at the subcortical level. The translation of emotional reactions into autonomic or endocrine outputs in the hypothalamus and brain stem is complex, and has received much attention. One of the areas of interest is the dorsomedial hypothalamus, which plays a key role in integrating acute psychological stress into cardiovascular responses. Stimulation of this center elicits cardiovascular responses similar to the fight-or-flight reactions [7]. Even more interestingly, inhibition of the center reduces heart rate and increases blood pressure in rats exposed to air jet stress [8].

Increased cardiovascular reactivity could also be an indicator of alterations in the peripheral tissue, like vascular wall thickening or altered receptor sensitivity. An otherwise normal central activation would lead to abnormal cardiovascular responses.

In our previous study [1], we found both exaggerated catecholamine responses as well as cardiovascular responses in subjects with high screening blood pressure. The concomitant increase in both cardiovascular responses and circulating catecholamines to MST, indicates that the hyperreactivity is more due to central mechanisms than alterations in peripheral tissue, i.e. increased sensitivity to catecholamines. Moreover, if there were peripheral mechanisms we would expect a similar hyperactivity to the physical stress tests given the same level of catecholamines in the three groups. Thus, it is reasonable to assume that the mechanism underlying the cardiovascular and sympathetic hyperreactivity is located in a cognitive-emotional or subcortical level, and may be associated with specific personality traits. We thus hypothesized in this study that a) stress reactivity is related to personality, and that b) responses to MST and CPT are influenced by different personality traits.

## Methods

### Subjects

All 19-year-old men in Norway have to attend a medical examination for the military draft procedure. The draft procedure usually takes a whole day and includes a psychological test, a test of physical strength and endurance, and a medical examination. Blood pressure measurements after 5 minutes sitting by means of an automatic auscultatory device were collected from all 19-year-old men attending in Oslo (N = 4137) during one year. Mean blood pressure was calculated as diastolic blood pressure + pulse pressure/3. The physicians were carefully instructed not to inform the subjects of the blood pressure reading. Therefore, they all got their final military physical fitness score and medical evaluation independent of the blood pressure recording. Rostrup et al have previously showed that awareness of hypertension induce higher detachment and lower impulsiveness, monotony avoidance, verbal aggression and irritability compared to unaware [9]. Giving such information to hyperreactive young men, thus, was a stressor that promoted detachment, a personality trait that was specifically investigated in the present study. Thus, hypertension labeling per se may be of significant importance.

We selected a total of 87 subjects from the screening, based on their mean blood pressure. 30 subjects belonged to the 1st percentile (70 ± 1 mmHg (mean ± SEM), Gr. 1), 30 to the 50th percentile (96 ± 2 mmHg, Gr. 2) and 27 to the 99th percentile of mean blood pressure (120 ± 1 mmHg, Gr. 3). There was an overrepresentation of hypo- and hypertensives, as regression to the mean may lead to insufficient dispersion in the laboratory. All were previously healthy without any history of diabetes, renal disease, elevated blood pressure or other cardiovascular disease. They had a normal physical examination and normal electrocardiogram, routine blood tests and urinalysis. None was on medical treatment or abused drugs or alcohol.

### Protocol

The study was approved by the local Ethics Committee, and informed consent was obtained from each subject selected from the screening. All subjects were examined by the same physician and only one subject each day. The physician was unaware to which group the subjects belonged. The examination started 08:00 a.m. following an 8-hour fast and at least 8-hour abstaining from nicotine and caffeine, and 24 hours abstaining from alcohol. Heart rate andblood pressure were recorded after 15 minutes at rest insitting position, by the same automatic auscultatory device as used atscreening.

A short teflon catheter (Venflon^®^, 19G, Viggo AB, Hälsingborg, Sweden) was introduced under local anaesthesia without epinephrine (Xylocain^®^, AstraZeneca) into the left brachial artery for blood sampling and intraarterial pressure monitoring as previously described [10].

Half of the participants rested supine for 30 minutes in the presence of the examining physician only. Intraarterial blood pressure and electrocardiogram were recorded continuously. At the end of this 30-minute period they were told of the CPT. The right hand was then completely immersed in ice water (0°C) for one minute. Thereafter, the subjects rested for 30 minutes before MST was announced. The subjects were asked to mentally subtract the number "13" repetitively for 5 minutes starting with "1079". A metronome making noise at a frequency of 2 Hz was used to distract the subjects. They were informed about any miscalculation. The other half did the tests in the opposite sequence.

Arterial blood for catecholamine assay was collected into polypropylene syringes after 30 minutes of supine rest, after 1/2 and 1 minutes of CPT, after 1, 3 and 5 minutes of MST, and after 5, 15 and 30 minutes of each recovery period.

Blood samples were immediately mixed with glutathione and EGTA, placed on ice, centrifuged at 4°C and the plasma frozen at -70°C until measurements of the catecholamines within a few weeks. A technician helped with blood sampling during the MST, and left the room thereafter. In all other parts of the study the subject and the physician were left alone.

### Assays

Blood was drawn in 10 mL glass tubes containing glutathione and EGTA, and plasma catecholamines were measured by a radioenzymatic technique according to Peuler and Johnsen [11] as previously reported [10,12]. On all samples, the same technician performed the assay examiner-blind.

### Karolinska Scale of Personality

To assess the different personality traits, we used the Karolinska Scale of Personality (KSP). KSP is a questionnaire comprising 135 questions, where each question is presented as a statement with a four-point response format ranging from 'does not apply at all' (= 1) to 'applies completely' (= 4). The items are grouped into 15 scales, and the values are summarized for each personality trait: Somatic anxiety (bodily signs of heightened anxiety such as restlessness and tachycardia), muscular tension (muscular tenseness and aches, difficulties in relaxing), psychic anxiety (worrying and low self-confidence), psychasthenia (low degree of mental energy and stress susceptible), inhibition of aggression (low assertiveness), impulsivity (tendency to act on impulse), monotony avoidance (experience-seeking behavior), detachment (no need for close relationships), socialization (relation with the respondent and his/her parents and other significant others during childhood), social desirability (conforming to social rules), indirect aggression (e.g. slamming doors), irritability (being easily annoyed, quick to anger), verbal aggression (e.g. shouting, quarrelling, cursing), suspicion (distrustfulness, projecting hostility to others), and guilt (feeling of remorse and shame) [13]. KSP was constructed primarily on theoretical consideration as a research tool to examine the biological basis of personality traits [14]. A longitudinal study over 9 years reported clear evidence of stability over time [15].

### Statistics

The data were analyzed using the statistical package SPSS version 12.0 for Windows (SPSS Inc, Chicago, IL). Parametric tests were used for normally distributed data and non-parametric when normality was not achieved by natural log transformation.

We analyzed possible independent explanatory predictors for cardiovascular and catecholamine variables at baseline and during stress in multiple linear regression analyses using a stepwise forward procedure. The independent variables were included in the model if p ≤ 0.2 in univariate analysis, and the dependent variables were not allowed to have inter-correlations with an r > 0.7. To create more separation between groups with different KSP scores, the subjects were divided into quartiles based on the score of the 15 KSP traits, with subsequent Chi-Square testing using linear-by-linear association to detect any relations with cardiovascular or catecholamine variables during rest and stress.

As a measurement of cardiovascular and catecholamine responses during stress, we used percentage change from baseline to mean stress. Data are presented as mean ± standard error of mean unless indicated otherwise, and null hypotheses were rejected if two-tailed p < 0.05.

## Results

### Baseline

Baseline characteristics are given in Table 1. By multiple regression analyses, personality traits turned out to be significant explanatory variables for baseline systolic blood pressure (positively related to suspicion), heart rate (positively related to somatic anxiety and negatively related to guilt and irritability) epinephrine (negatively related to inhibition of aggression) and norepinephrine (positively related to suspicion) (Table 2).

**Table 1:** Baseline and stress characteristics

**Variables (N = 87)**	**Baseline**	**Mental stress test**	**Cold pressor test**
Systolic BP (mmHg)	125.0 ± 1.5	148.5 ± 2.1	145.1 ± 1.6
Diastolic BP (mmHg)	65.2 ± 0.9	79.5 ± 1.1	80.5 ± 1.0
Heart rate (beats/min)	60.7 ± 0.9	80.8 ± 1.8	69.4 ± 1.2
Epinephrine (pg/mL)	45.22 ± 3.39	119.3 ± 8.9	78.3 ± 5.2
Norepinephrine (pg/mL)	110.70 ± 6.14	198.0 ± 9.3	158.5 ± 8.4
Total cholesterol (mmol/L)	4.01 ± 0.08		
HDL (mmol/L)	1.12 ± 0.02		
HDL:total cholesterol ratio	0.29 ± 0.01		
Triglycerides (mmol/L)	0.84 ± 0.04		
Fasting glucose (mmol/L)	4.21 ± 0.06		
Body mass index (kg/m2)	22.39 ± 0.32		

The values are given as mean ± SEM. BP: blood pressure, HDL: high density lipoprotein.

**Table 2:** Multiple regression analyses of baseline variables

**Dependent variables (R^**2 **^and p for the model)**	**Model**	**β**	**P**
Baseline DBP (R^2 ^= 0.180, p = 0.001)	Baseline norepinephrine	0.346	0.003
	BMI	0.298	0.010
Baseline SBP (R^2 ^= 0.135, p = 0.008)	Baseline epinephrine	0.300	0.011
	Suspicion	0.230	0.049
Baseline HR (R^2 ^= 0.219, p = 0.001)	Somatic anxiety	0.474	<0.001
	Guilt	-0.289	0.022
	Irritability	-0.257	0.043
Baseline epinephrine (R^2 ^= 0.060, p = 0.042)	Inhibition of aggression	-0.244	0.042
Baseline norepinephrine (R^2 ^= 0.063, p = 0.037)	Suspicion	0.250	0.037

SBP/DBP: systolic/diastolic blood pressure; HR: heart rate; BMI: body mass index.

**Figure 1:**
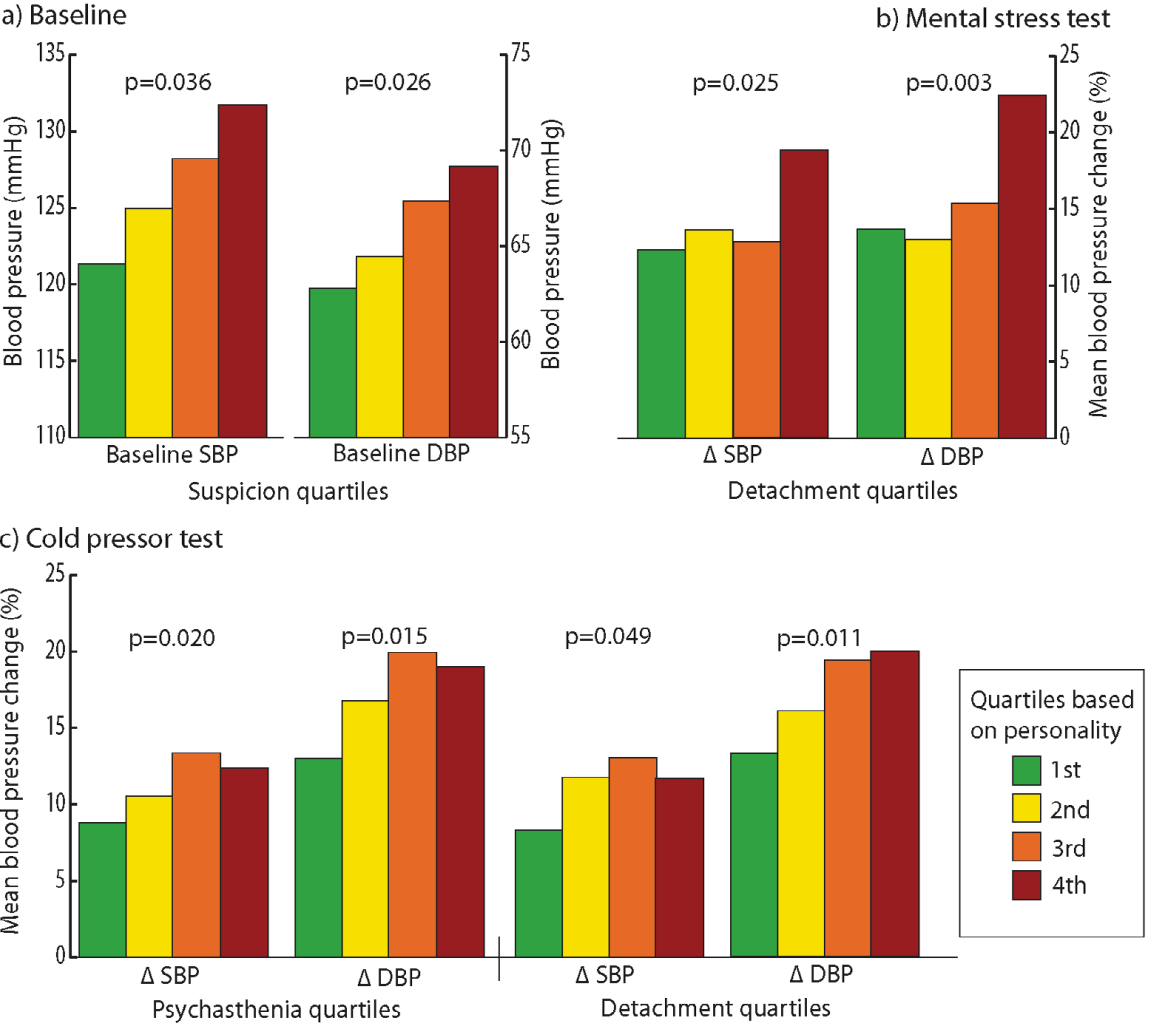
**Blood pressures at baseline and changes during stress tests in the personality quartiles**. Illustration of blood pressures at baseline and changes during stress in the personality quartiles (Chi-Square test with linear-by-linear association). Suspicion at baseline (a), detachment during mental stress test (b) and psychasthenia and detachment during cold pressor test (c) were the only personalities that showed significant dependencies to blood pressure levels. SBP/DBP: systolic and diastolic blood pressure.

When we divided the subjects into quartiles based on the KSP score, suspicion was the only personality trait that showed a significant association with systolic (p = 0.036) and diastolic blood pressure (p = 0.026) (figure 1a) and norepinephrine (p = 0.036) at baseline.

### Stress reactivity and personality

Mean cardiovascular and catecholamine responses during stress tests are shown in table 1. Results from the multiple regression analyses of the responses during stress (Table 3), show that the psychological variables detachment and verbal aggression were significant predictors of diastolic blood pressure and norepinephrine response to MST, respectively. During CPT, the level of psychasthenia, detachment, somatic anxiety, irritability and muscular tension were significant explanatory variables for both cardiovascular and catecholamine reactivity.

**Table 3:** Forward stepwise multiple regression analyses of cardiovascular and catecholamine responses to stress

	**Dependent variable (R^**2 **^and p for the model)**	**Model**	**β**	**P**
MST	Δ DBP (R^2 ^= 0.262, p = 0.002)	Mean epinephrine during MST	0.334	0.009
		Baseline DBP	-0.319	0.013
		Detachment	0.253	0.044
	Δ SBP (R^2 ^= 0.317, p < 0.001)	Mean epinephrine during MST	0.551	<0.001
		Baseline SBP	-0.345	0.004
	Δ HR (R^2 ^= 0.261, p < 0.001)	Mean epinephrine during MST	0.511	<0.001
	Δ epinephrine (R^2 ^= 0.142, p = 0.004)	Baseline epinephrine	-0.376	0.004
	Δ norepinephrine (R^2 ^= 0.285, p < 0.001)	Baseline norepinephrine	-0.465	<0.001
		Verbal aggression	-0.252	0.031
CPT	Δ DBP (R^2 ^= 0.303, p < 0.001)	Baseline DBP	-0.506	<0.001
		Psychasthenia	0.234	0.031
	Δ SBP (R^2 ^= 0.258, p < 0.001)	Baseline SBP	-0.396	0.001
		Detachment	0.272	0.017
		Somatic anxiety	0.225	0.046
	Δ HR (R^2 ^= 0.210, p = 0.001)	Baseline HR	-0.309	0.009
		Irritability	0.282	0.016
	Δ epinephrine (R^2 ^= 0.186, p < 0.001)	Baseline epinephrine	-0.432	<0.001
	Δ norepinephrine (R^2 ^= 0.437, p < 0.001)	Baseline norepinephrine	-0.571	<0.001
		Muscular tension	0.298	0.004

Δ: delta; MST: mental stress test; CPT: cold pressor test; SBP/DBP: systolic/diastolic blood pressure; HR heart rate.

Based on KSP-traits quartiles, delta (Δ) systolic (p = 0.025) and Δ diastolic blood pressure (p = 0.003) during MST were related to detachment score, with the highest reactivity in the 4^th ^quartile (Figure 1b), while Δ norepinephrine was significantly related to muscular tension (p = 0.033). No other personality traits showed significant relations during MST. Responses to CPT were associated with three personality traits: Δ systolic and Δ diastolic blood pressure were related to detachment (p = 0.049 and p = 0.011, respectively) and psychasthenia (p = 0.020 and p = 0.015) (Figure 1c), while subjects characterized by high verbal aggression had lower response to CPT as measured by Δ norepinephrine (p = 0.037).

## Discussion

The purpose of the present study was to examine whether responses to MST and CPT are influenced by personality, and identify possible personality traits related to increased reactivity. Our findings indicate that personality is related to cardiovascular and catecholamine levels both at rest and during stress. When the subjects were divided into quartiles based on the various personality scores, only suspicion was associated with resting values. There was no single personality trait that explained most of the stress response. However, detachment was related to both MST and CPT responses. Reactivity during MST was related to the level of detachment and muscular tension, while responses to CPT were influenced by detachment, psychasthenia and verbal aggression.

Blood pressure and catecholamine levels at rest and during stress tests represent two different kinds of physiological states. One may consider blood pressure as having two components – a basal or tonic level, and phasic fluctuations around this [16]. Reactivity testing is a method to examine the latter, which is believed to have a predictive value of cardiovascular disease independently of the resting state.

Cardiovascular reactivity has been associated with a new personality construct, the type D or distressed personality [17]. Type D is defined as the interaction of a) negative affectivity, with a tendency to experience negative emotions, including depressed mood, anxiety, anger, and hostile feelings, negative view of self and more somatic symptoms, and b) social inhibition, characterized by avoidance of potential dangers involved in social interactions and feeling inhibited, tense, uncomfortable and insecure. Several of the traits that were significant predictors of reactivity in our study support this model; detachment, muscular tension, somatic anxiety, psychasthenia and irritability.

Detachment was the only trait that was related to both MST and CPT reactivity. This trait is characterized by a need to remain distant, and a fear of closeness, and it is regarded as the opposite of attachment in the closeness-separateness dimension [18]. Social phobia is among other variables, associated with high scores in detachment [19]. During the stress tests, the subjects are forced into a setting where they have to perform. This is not desirable for a person who has a fear of closeness, and may elicit exaggerated responses. Schalling et al [20] has classified the KSP scales into 5 factors based on factor analysis, where detachment constitutes together with impulsiveness and monotony avoidance the factor of intro-/extroversion. Arkwright et al [21] has documented that introverted non-smoking moderate to heavy alcohol drinkers have a prevalence of hypertension three times that of extroverted drinkers. Furthermore, an inverse relation between extroversion and systolic blood pressure is described among untreated elderly women [22]. However, we are the first, to our knowledge, to demonstrate a positive relationship between detachment and cardiovascular reactivity.

Inhibition of emotions has been associated with higher cardiovascular reactivity [3], lower cardiovascular recovery [23], lower heart rate variability [24] and in the long term, carotid atherosclerosis [25], incidence of coronary heart disease [26] and cardiac mortality [27]. This inhibition may lead to a canalizing of feelings into somatic reactions. Theorell et al [28] examined young men with low, normal and high blood pressure and compared type A behavior and the KSP scales for impulsivity, indirect aggression and verbal aggression. They discovered a lower impulsivity score in the high blood pressure group compared with the two other groups, and they also reported that low scores of impulsivity and verbal and indirect aggression were associated with higher systolic blood pressure. This is in accordance with the present study where an inverse relation between verbal aggression and reactivity during MST was demonstrated, while "inward" personality traits like detachment, psychasthenia, muscular tension and somatic anxiety was positively related to reactivity. This supports the hypothesis that some personalities are characterized by a suppression of feelings in a way that raises the blood pressure, while those who express their feelings to the surroundings is more likely to have lower blood pressure and reactivity.

We had prior to the study expected that the psychological test MST would be more influenced by personality than the physical test CPT. However, CPT is not a pure physical test, and may involve other psychological elements like pain, expectations, fear, motivation and irritability. These factors may explain why many personality traits turned out to be important predictors of CPT reactivity.

The fact that we found increasing resting blood pressure with increasing suspicion score makes sense. The subjects did not know what to expect from the coming examination, and a high level of suspicion may have influenced the blood pressure due to a lack of control in the laboratory setting. Borderline hypertensives have been described as being more hostile and suspicious [29]. Others have shown a negative relation between suspicion and systolic blood pressure among elderly [22]. Suspicion is a part of the KSP-based factor of hostility, together with guilt. As the most predictive characteristic of type A behavior for poor health outcome, hostility is a well known risk factor [30]. Several authors have reported a relationship between hostility and hypertension and cardiovascular reactivity [31-33].

In the current study, suspicion did not turn out to be an explanatory variable for stress reactivity, unlike in the resting condition, indicating that different personality traits may have different impact of cardiovascular regulation at rest and during stress. This is further supported by the fact that irritability was negatively related to resting heart rate and positively related to heart rate during stress.

The multiple regression analyses demonstrated that the various reactivity variables (BP and catecholamines) were negatively predicted by their baseline variables. This finding may seem unlikely, but is believed to be a statistical phenomenon, where a higher baseline value involves a lower potential to increase during stress.

Our results should be interpreted cautiously. In a cross sectional study it is impossible to state whether personality influences stress reactivity or the opposite. A statistical relation does not mean that there is a causal relationship. Although the KSP covers a wide range of personalities, one may question whether the questionnaire comprises the traits that are important in a fight-or-flight situation. As the KSP constitutes 15 variables, and we take several blood pressure and catecholamine measurements during the session, multiple correlation analyzes may give us some false positive results. The statistical power to detect differences was limited because of the relatively small number of subjects. Despite these limitations, the findings were consistent. Confounding factors were eliminated to a minimum in our study, as all subjects were Caucasian men at the same age with no medication and similar body mass index. The population in Oslo, as in Norway in general, is stable, thus is genetically homogenous. Arterial catecholamines assessed in the study is a better estimate of overall sympathetic activation compared to venous [12]. All the subjects in the current study were unaware of their blood pressure level, thus avoiding the potential confounding factor of awareness. In a previous study, there were no personality differences between unaware hypertensives and normotensives, while aware hypertensives scored higher on neuroticism, trait anxiety, state anxiety, state anger and type A behavior [34].

## Conclusion

The present study demonstrates how certain personalities may be a threat against health and well-being, as exaggerated cardiovascular responses to stressful situations are well known predictors of future morbidity and mortality [35]. Our findings indicate that cardiovascular and catecholamine stress reactivity is dependent on different personality traits, without any single trait being clearly dominant Furthermore, personality seems to have as much, or perhaps more importance in predicting responses to CPT than responses to MST.

## Competing interests

The author(s) declare that they have no competing interests.

## Authors' contributions

AF carried out the statistical analyses and drafted the manuscript. ØE participated in the design of the study, helped with statistical analyses. SEK helped to draft the manuscript. MR conceived of the study, and participated in its design and coordination and helped to draft the manuscript. All authors read and approved the final manuscript.
